# Hemodialysis Vascular Access: A Historical Perspective on Access Promotion, Barriers, and Lessons for the Future

**DOI:** 10.1016/j.xkme.2024.100871

**Published:** 2024-07-15

**Authors:** Anatole Besarab, Stanley Frinak, Suresh Margassery, Jay B. Wish

**Affiliations:** 1 Department of Medicine, Stanford University School of Medicine, Stanford, CA; 2 Department of Medicine, Henry Ford Health System, Detroit, MI; 3 Dallas Vascular Center, Dallas, TX; 4 Department of Medicine, Indiana University School of Medicine, Indianapolis, IN

**Keywords:** Catheter, fistula, graft, hemodialysis, vascular access

## Abstract

This review describes the history of vascular access for hemodialysis (HD) over the past 8 decades. Reliable, repeatable vascular access for outpatient HD began in the 1960s with the Quinton-Scribner shunt. This was followed by the autologous Brecia-Cimino radial-cephalic arteriovenous fistula (AVF), which dominated HD vascular access for the next 20 years. Delayed referral and the requirement of 1.5-3 months for AVF maturation led to the development of and increasing dependence on synthetic arteriovenous grafts (AVGs) and tunneled central venous catheters, both of which have higher thrombosis and infection risks than AVFs. The use of AVGs and tunneled central venous catheters increased progressively to the point that, in 1997, the first evidence-based clinical practice guidelines for HD vascular access recommended that they only be used if a functioning AVF could not be established. Efforts to promote AVF use in the United States during the past 2 decades doubled their prevalence; however, recent practice guidelines acknowledge that not all patients receiving HD are ideally suited for an AVF. Nonetheless, improved referral for AVF placement before dialysis initiation and improved conversion of failing AVGs to AVFs may increase AVF use among patients in whom they are appropriate.

Many barriers to optimal health care delivery are due to decreased investment in new systems and technologies, a shrinking workforce, discrepancies in health care accessibility, and reduction of government support for research and clinical care. Such barriers are particularly evident in the transition of patients with advanced stages of chronic kidney disease (CKD) to dialysis. CKD disproportionately affects underserved populations, such as racial minorities and those with lower socioeconomic status who often live in inner-city neighborhoods and rural areas. Such individuals frequently have poor access to health care providers because of inadequate health insurance, long travel times, language barriers, and lack of tools to navigate the health care system. Provision of health care to these patients is often on a sporadic as-needed basis. CKD, which requires serum creatinine levels and the urinary albumin-creatinine ratio for staging and risk stratification, is underdiagnosed and undertreated. Referral to a nephrologist for treatment that may slow CKD progression and/or prepare the patient for kidney replacement therapy (KRT) may be late or nonexistent.^1^ As a result, almost half of incident KRT patients in the United States “crash-land” to dialysis treatment with no medical or educational preparation, especially with regards to KRT modality options and HD vascular access. Between 2014 and 2020, dialysis facilities serving majority Black ZIP code tabulation areas or ZIP code tabulation areas with median annual income <$45,000 achieved significantly lower arteriovenous fistula (AVF) rates (*P* < 0.05) than their non-Black, higher income counterparts.^2^ The trend toward initiation of dialysis with higher levels of residual kidney function has also made it more challenging to time placement of an AVF so it is functional when needed. Between 1996 and 2012, the percentage of patients starting dialysis with an estimated glomerular filtration rate (eGFR) of 10-14.9 mL/min increased from 9% to 27%, and the percentage of those with eGFR >15 mL/min increased from 3% to 13%.^3^ For those patients with CKD who are fortunate to receive education regarding KRT modality options, consideration of and evaluation for peritoneal dialysis may postpone the placement of vascular access among patients whose KRT is ultimately initiated while the patient is receiving HD.^4^

The National Academies of Sciences, Engineering, and Medicine (NASEM) Work Group developed a “scorecard” as a baseline for future evaluations of primary care^5^ and recommends a stronger investment in high-quality primary care, the foundation of the health care system. The report emphasized the essentiality of preventive care and early interventions. The NASEM report concluded that the US health care system is in critical need of change. The barriers to optimal preparation for KRT are a microcosm of problems in the US health care system, and the solutions proposed by the NASEM align with those for improving preparation of patients for KRT. The NASEM committee provided the following 3 recommendations for the overall system that also apply to improving the timely establishment of vascular access for HD:

• Establish and train *interdisciplinary teams* to function where people live and work.
• Pay such interdisciplinary teams to *care for people*, rather than continue to deliver services via a fee for service model.
• Design *information technology* that serves the patient, the family, and the interdisciplinary care team.

The problem of delayed creation of permanent vascular access for HD reflects the ongoing conflict between systems, ie, those who are delegated to do it, including nephrologists, surgeons, and dialysis organizations, and those who pay for it, namely, the Centers for Medicare and Medicaid Services (CMS) and private insurers. Since the implementation of the end-stage renal disease (ESRD) program in 1973,^6^ policies have not always provided the best balance of benefit to payors, providers, and patients.

This paper focuses on the history of vascular access, describing the past and present barriers to the implementation of timely vascular access for HD. Special emphasis is placed on the “failure” of the autologous AVF to maintain its 20-year dominance as the preferred permanent access for HD after the mid-1980s. More frequent use of synthetic arteriovenous grafts (AVGs) and dependence on tunneled central venous catheters (CVCs) has significant risks. We believe that the use of tunneled CVC even for initiation of HD, while waiting for AVF maturation, should be one of “last resort” as advocated since 1997 by evidence-based clinical practice guidelines.^7^, ^8^, ^9^, ^10^

## Historical Background and Perspective

**Figure 1:**
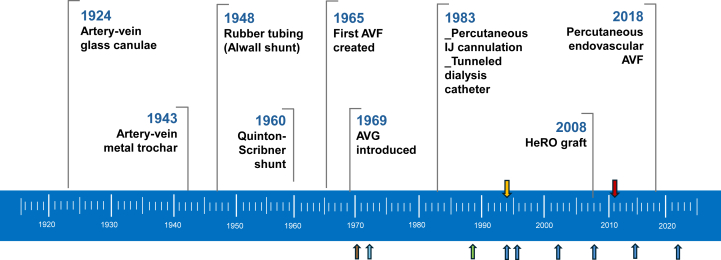
Timeline of hemodialysis vascular access. Dark blue arrows represent the timing of NKF-KDOQI vascular access clinical practice guidelines. Brown arrow reflects the formation of National Medical Care, the first for profit dialysis provider. Light blue arrow points to passage of 1972 Public law 92-603, section 2991 authorizing Medicare payments for treatment of patients with kidney failure. Green arrow reflects establishment of the U.S. Renal Data System. Orange arrow reflects establishment of ESRD Clinical Performance Measures Project. Red arrow reflects onset of bundled payment for dialysis. Abbreviations: PTFE, polytetrafluoroethylene; AVF, arteriovenous fistula; AVG, arteriovenous graft; TDC, tunneled dialysis catheter; HeRO, hemodialysis reliable outflow graft. Figure adapted from Hassanein M et al.^46^

**Figure 2:**
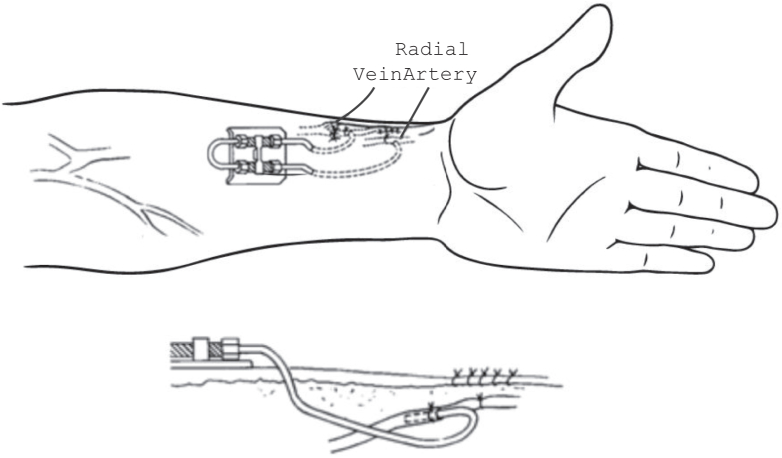
The Quinton-Scribner shunt.

Figure 1 shows the timeline for vascular access development over the past 80 years. Initially, vascular access for HD used glass cannulas or metal trocars. During and after World War II, Kolff^11^ used these to treat acute kidney injury in trauma patients. A shunt, developed in Seattle by Quinton et al^12^ in the early 1960s, permitted multiple HD sessions using the same access. The Quinton-Scribner shunt, most often placed at the wrist (Figure 2) as well as the ankle, allowed life sustaining extended maintenance dialysis therapy.

Few centers could match Belding Scribner's early successes. Lundberg et al^13^ reported their experience in 56 patients (mean age, 39 years) who required 236 Scribner shunts for intermittent HD in the years 1965-1971. The mean shunt survival time was 11.2 months for the arterial and 9.0 months for the venous component. The shunt clotting rate was 0.6 episodes/month over 96.4 patient-years. Failure of saline flushing to resolve shunt thrombosis mandated streptokinase thrombolysis (successful in 80% of instances); later, the Fogarty balloon aided in thrombus removal. Shunt cannula infection was common. Mild-to-severe bleeding was a complication of anticoagulant therapy among patients with repeated clotting of the device.

## Growth of the ESRD Program

The Health Care Financing Administration and its successor CMS noted that patients receiving KRT consumed a disproportionate amount of financial and human resources costs because the Medicare ESRD Program grew at a rate of 8.76% per year over the first 2 decades following its inception in 1973.^14^ Approximately 218,000 patients received KRT in 1991 (2.75-fold higher than the plateau estimated in 1973) with almost 50,000 being incident patients. The increase was most dramatic among African Americans and American Indians. The first-year gross mortality of 22% was insufficient to prevent growth of the population requiring KRT. Of the factors noted to be contributory to mortality, only age and sex were immutable. Sex is defined here as the biologic identity of the individual, male or female.

Overall, first-year mortality, about 20%-22%, did not change for the first 18 years of the ESRD program.^14^, ^15^, ^16^, ^17^ However, neither Health Care Financing Administration/CMS administrators nor the dialysis organizations factored in the increase in mean age from 49.9 to 61 years or recognized that a stable gross mortality rate in the face of an increasing age and obesity was a success. Since 1990, the population receiving KRT has continued to progressively age with a large increase in the fraction of patients having diabetic nephropathy and other comorbid conditions.

When the dialysis dose (Kt/V) was increased nationally in the United States in the 1990s, crude mortality rates fell from 21.8 before 1990 to 19.5% in the mid-late 1990s. When adjusted for various risk factors, the standardized mortality was reduced to 0.75^18^ between the 2 periods. During the latter part of the same decade in which the ESRD Clinical Performance Measures Project was in effect, gradual improvement in crude mortality continued to accrue.^19^ As of 2019, all-cause mortality has declined over to all-time low for US patients requiring KRT. Whether mortality rate could be lowered to those reported by some EU nations and Japan is questionable because US patients are older and have higher degrees of comorbid conditions.

Continued growth of the population receiving KRT increased overall costs by 57% between 1999 and 2004*,* constituting 6.7% of total Medicare expenditures. This evolution of the ESRD Program was associated with diminished per-treatment reimbursement in inflation-adjusted dollars, a dramatic increase in costs associated with medications administered during dialysis (erythropoiesis-stimulating agents, intravenous iron, and vitamin D analogs), a steady decline in the use of home dialysis (including peritoneal dialysis), and further consolidation of the for-profit dialysis provider industry.^20^

## Evolution of Vascular Access

Vascular access for HD evolved in parallel with the events described above. The first major advance away from the Quinton-Scribner shunt was the development of the Brescia-Cimino-Appel-Hurwich radio-cephalic AVF (RC-AVF) in the mid to late 1960’s,^21^ which revolutionized vascular access for the next 2 decades. The AVF matures slowly with access blood flow increasing for up to 3 months. Kolff^11^ advocated use of a small gauge single needle for initial canulation and dialysis because of the lower intra-access blood flow and recommended a 4-week period for maturation. It was possible (in most cases), with single needle cannulation, to achieve extracorporeal blood flows of 200-250 mL/min.

Advantages of single needle dialysis include fewer punctures of small or maturing access systems and avoidance of central vein catheter use. Because of the low-mass transfer coefficients of the dialyzers used in the 1970s to early 1980s, longer dialysis duration (5-6 hours) was practiced and was sufficient to attain Kt/V values ≥1.2 despite some degree of recirculation. The typical dialysis session with single needle dialysis was 4-6 hours in the late 1970s. After the fistula matured for an additional 4-8 weeks, most AVFs tolerated 2 needle cannulations, delivering pump blood flows of 250-350 mL/min. Following publication of the National Dialysis Cooperative Study in 1981,^22^ average treatment times of 4-4.5 hours were considered adequate.

With the development of larger high-efficiency/high-flux dialyzers and the prescription of higher blood flows (350-500 mL/min), dialysis treatments became progressively shorter. Shorter treatments allow more shifts of patients per day at a single station. Patients also favor shorter treatment times. Long-term HD therapy, as practiced in the United States in the early 1990s, consisted of 3 sessions per week, each 3-4.5 hours in duration.^23^ During this period, reports appeared on commuting time to/from dialysis^24^ and postdialysis “washout”.^25^ Duration of “washout” and poorer quality of life (QoL) measures were found to be linearly associated with increased risks of death and hospitalization.^26^ Importantly, both decreases in blood pressure and feeling washed out or drained were identified by patients as more important outcomes than death or hospitalization.^27^

Shortening of dialysis treatment time was ultimately shown not to benefit patients.^27^ Patients receiving an average dialysis treatment duration of <3.5 hours had relative mortality risks of 1.17-2.18 compared with those with treatments longer than 3.5 hours.^15^ With shorter treatment times, ultrafiltration rates increased, leading to more frequent episodes of intradialytic hypotension (IDH), ranging from 5%-40% of treatments.^28^ More current rates of IDH are about 8%-10% of treatments^29^ that meet the Kidney Disease Outcomes Quality Initiative (KDOQI) definition.^30^ The incidence of IDH is associated with increased risk of vascular access thrombosis.^31^

Treatment times have gradually increased over the past 10-15 years, but blood flow rates currently prescribed are still higher than they need to be to achieve Kt/V ≥1.4^32^ and on average 50-100 mL/min greater than those prescribed in other nations (250-350 mL/min). Studies indicate that more frequent and/or longer dialysis improves QoL, controls hyperphosphatemia, reduces hypertension, and leads to regression of left ventricular hypertrophy,^25^^,^^33^ particularly with long dialysis treatments (7-8 hours overnight). However, most patients prefer conventional 3.5-4.5 hour duration HD over longer nocturnal treatments.

Bovine and then synthetic AVGs were introduced in 1969, 3 years after the Brecia-Cimino AVF, for those individuals without adequate arterial or venous anatomy for a native AVF. Tunneled CVCs were introduced in the 1980s.

In 1996, Feldman et al^34^ presented data on the magnitude and growth of vascular access-related hospitalization in the United States. Total costs of the morbidity associated with AVFs, polytetrafluoroethylene AVGs, and tunneled CVCs was expected to crest >$1 billion per year. They noted that medical practice was evolving away from the use of RC-AVFs in favor of AVGs and AVF construction at or above the elbow instead of forearm. Although the appropriate roles for brachial AVF, AVG, and tunneled CVCs 28 years ago were uncertain, complications associated with both AVGs and tunneled CVCs relative to RC-AVF were higher. These authors advocated strategies to detect vascular access dysfunction, especially in AVGs, and to better identify those patients in whom RC-AVF was still a realistic access option.

Efforts to improve vascular access outcomes in the US were launched after the National Kidney Foundation Dialysis Outcomes Quality Initiative (DOQI) Clinical Guidelines for Vascular Access were published 1997.^7^ Kidney Disease Outcomes Quality Initiative (KDOQI) Guidelines for Vascular Access were updated in 2000,^8^ 2006,^35^ and 2019.^9^ National dialysis vascular access quality improvement programs were implemented in the United States; Fistula First Improvement Initiative (2003) became the Fistula First Breakthrough Initiative (2005), followed by the Fistula First-Catheter Last initiative (2015). Unfortunately, none of these initiatives produced the desired outcomes of functional AVF within the aspirational maturation time of 3-4 months, despite an increase in AVF prevalence to a target of 65%.^36^ At HD initiation in the United States in 2010-2011, mature AVF constituted 16.2% of accesses, tunneled CVC 80.8%, and AVG 3.0%. Among those initiating HD with a tunneled CVC, only 42.7% transitioned to a permanent AVF or AVG access by 6 months. Compared with an AVF that matured and maintained primary patency in year 1 at an average cost of $7,871 per patient, vascular access costs in the first year were 2.25 greater in patients whose AVFs did not mature and 4.01 greater in those who experienced secondary AVF patency loss or whose AVF was abandoned without use.^36^

In the above analysis, factors such as aging of patients with kidney failure and the growing influence of obesity on access choice and maturation were not assessed. A “ray of sunshine” was the observation that the rate of starting dialysis with a functional AVF was much higher at 29.7% compared with 5.1% among patients with prior nephrology care for >12 months. This led to adoption of the “right access for the right patient at the right time concept” in the 2019 updates of the KDOQI vascular access guidelines.^8^^,^^9^^,^^37^

## Barriers to Improving AVF Results in the United States

US nephrologists and vascular surgeons defend our “poorer” performance than other developed world nations (DWNs) citing many factors. There are no restrictions on eligibility for KRT in the Unites States; access to dialysis is covered by Medicare/Medicaid and private insurance. Patients initiating dialysis in the United States are also older and have more comorbid conditions. Differences in vascular health of patients receiving HD among global/ethnic populations do exist and affect the choice for AVF versus AVG. Obesity, diabetes, and greater severity of cardiovascular and peripheral vascular disease is more rampant in the United States compared with other DWNs. Our past attempts to promote AVF in US population with greater degrees of atherosclerotic vascular disease than DWNs may explain why the United States achieved a higher fraction of AVF construction but a poorer rate of AVF maturation and abandonment than other DWNs.

Prolonged use of tunneled CVCs is associated with more infections, thus increasing costs. The 2019 update of the KDOQI vascular access guidelines^9^^,^^10^^,^^37^ appropriately took a more nuanced view to individualize the choice of vascular access based on patient factors such as prognosis and vascular suitability. This is a preferred patient-centered rather than a “one size fits all” approach. The best access for a particular patient may be an AVG or even a tunneled CVC rather than an AVF, especially in the elderly and those with limited life expectancy. Viecelli and Lok^38^ advocate a patient-centered coordinated multidisciplinary approach that aligns the patient’s End-Stage Kidney Disease Life-Plan (individualized treatment considerations of patient preferences, likelihood of vascular access function and survival and potential complications in the context of available resources and life expectancy) with the most suitable vascular access. This approach is echoed by Kalloo et al^39^ who note that patients not suitable for an AVF may include the elderly and those with limited life expectancy as well as those with poor vascular anatomy and with slowly progressive CKD who are more likely to die than progress to end-stage kidney disease. These authors bemoan the “one size fits all” design of the CMS ESRD quality incentive program (QIP) that financially penalizes dialysis providers for low AVF and high-tunneled CVC rates without considering patient-specific factors that may affect vascular access choice. In doing so, patients may be steered to a vascular access type that leads to numerous unnecessary surgical and interventional procedures with minimal to no gains in clinical outcomes and decrease in QoL. It is notable that the QIP has not achieved the stated goals of CMS to increase prevalent AVF rates >68% and reduce long-term (>90 day) tunneled CVC rates <10%.^2^

We believe that “relatively poor” results regarding AVF “success” in the United States is not just because of atherosclerosis or increased vascular calcification but the following 3 additional factors: (1) late referral of patients for access evaluation preventing timely access placement; (2) patient denial of their imminent need for dialysis within the forthcoming 6 months; and (3) inadequate surgical training in vascular access evaluation and construction.

## When to Plan Then Place AV Access and Start Hemodialysis

Optimal initiation of HD requires predialysis placement of permanent access directed by an orderly planned process, which provides sufficient lead time for maturation. Morbidity and mortality are lower in planned initiation of dialysis.

AVF creation should allow at least a 4-6–month period for adequate AVF maturation. Initial cannulations should use 17- or 16-guage needles before progressing to 15-guage needles; 14-gauge needles should be avoided because of trauma to the access and excessive postdialysis bleeding. We recommend that the patient be evaluated and AVF access placed as the eGFR approaches 10-15 mL/min/1.73 m^2^. Allon et al^40^ analyzed outcomes in 286 patients who had predialysis placement of an AVF. The median time from access surgery to HD initiation was 69, 156, and 429 days in patients with an eGFR of <10, 10-14 and >15 mL/min/1.73 m^2^, respectively.

Because AVGs are mature at implantation, a lead time of 10-14 days for standard bovine or polytetrafluoroethylene grafts is adequate. This interval permits graft incorporation by the surrounding connective tissue and resolution of any postoperative edema. A tight tunnel allows the graft to be cannulated in 1-3 days. Currently, more rapid cannulation of AVG made possible by expensive hybrid materials, which “self-seal” are a viable cost alternative to bridging with a tunneled CVC.^41^^,^^42^ Bioengineered vessels have been successfully used as arteriovenous conduits as well.^43^

The predialysis vascular access placement study by Allon et al^40^ also found that catheter-free HD initiation was higher in patients with an AVF (88%) than an AVG (48%) when the eGFR was <10-14 mL/min/1.73 m^2^. Patients receiving an AVF were more likely to undergo an angioplasty (11% vs 0%), surgical access revision (26% vs 8%,), catheter insertion (31% vs 11%) compared with those receiving an AVG, respectively (all differences *P* < 0.0001). A second access placement was also more frequent with AVFs (16% vs 6%, *P* = 0.02). To avoid a tunneled CVC at dialysis initiation, both optimal timing and better determinants for access type need to be developed.

## Converting AVG to AVF – Salvaging the Vein

Depending on the AVG anatomical arterial site, an AVF can also be constructed when an AVG shows signs of impending failure. Slayden et al^44^ reported their experience with secondary arteriovenous fistula (SAVF) using the existing mature AVG outflow vein. In 40 consecutive patients with previous interventions, 37 underwent SAVF surgery although the AVG was still patent. Cumulative SAVF patency was 92.5% and 87.5% at 1 and 2 years, respectively. Salvage after occurrence of AVG thrombosis was achievable in only 20 of 102 patients. However, in these 20 patients, SAVF cumulative patency was excellent with 94.4% at 1 year and 91.6% at 2 years. Acting before AVG thrombosis is key in achieving optimal results.

Salman et al^45^ also reported outcomes for SAVF performed in 62 patients over a 5-year period in those with a patent outflow vein (type 1, n = 35) or its absence (type 2, n = 27). For type 1 situations, SAVF produced cumulative secondary patency rates of 100% at 2 years and 84% at 3 years. For type 2 situations in which the vein was not in proximity to the original site, corresponding cumulative patency was 96% and 91% at 2 and 3 years, respectively. In either situation, mean number of procedures needed to maintain patency was 1.4-1.5 per year. Tunneled CVC was required in 60% of patients with type 1 and 100% of patients with type 2 SAVF. When timed properly, secondary cumulative patency with reasonable intervention rate in SAVF is as good as that reported for a primary AVF and without the issue of “failure to mature” if the vein is patent at the time of conversion.

From the viewpoint of patients and the health care system, the dramatic reduction in catheter dependency from a SAVF conversion could reduce costs and improve clinical outcomes. Most patients undergoing HD whose access is an AVG will have one or more anatomic sites and vessels suitable for an autogenous SAVF. Vessel mapping is critical in the evaluation of failing AVGs and in preparation for a SAVF. Performing conversions while a vein is patent virtually abolishes the need for tunneled CVC use after the conversion.

Both monitoring and surveillance are needed to permit appropriate interventions (endovascular or surgical) to reduce thrombosis risk, maintain patency, and preserve the opportunity for SAVF. Future studies are needed to determine when the critical point for converting an AVG is reached. Excellent 3-year patency can still be achieved^44^^,^^45^ even if the need for short term tunneled CVC cannot be avoided.

## Conclusions

Fifty years ago, the US ESRD Program was initiated and then emulated to various degrees worldwide, allowing survival for several million individuals.^46^ The ability to track outcomes among patients became robust with the establishment of the United States Renal Data System in 1988, which allowed collection and analysis of the most robust disease-specific data sets available within the Medicare population. Ultimately, studies of outcomes from this observational data set require sophisticated statistical tools and vetting in a peer review process before publication. These publications facilitated the development of quality goals and metrics, followed by evidence-based clinical practice guidelines. In 2003, CMS and other stakeholders developed a national quality improvement effort to increase the use of AVFs as the preferred choice for vascular access. This collaborative initiative, known as Fistula First and its successors, led to a dramatic increase in the construction of AVFs, of which up to 40% failed to mature. Reasons for maturation failure include increasing age of the population of incident kidney failure requiring KRT, more severe peripheral arterial disease, diabetes in almost half of all incident patients, and an epidemic of obesity.

Many aspects of creating the right access for the right patient at the right time need to be implemented on a more widespread scale. These include more thorough Doppler ultrasound evaluations to select the best artery–vein site, novel endovascular devices for creation of AVFs, use of bioengineered grafts, and SAVFs. Fistula First dramatically increased the prevalence of AVFs in the United States because it was a collaborative quality improvement program that offered educational tools to stakeholders. The current QIP system is a one size fits all provider payment penalty that may punish the facilities that treat the most socioeconomically disadvantaged patients with the fewest resources to improve vascular access outcomes. The AVF prevalence rate in the United States may well have “topped out” at 64%-68% (Table 1) when patient-centered limitations to AVF use are considered.^2^ The goals of vascular access should now be increasing incident AVF rates with more timely, targeted (the right vessels in the right patient) vascular access placement among patients with advanced CKD not yet requiring KRT; acknowledging that a majority of AVFs will require more than one surgical procedure; prolonging the functional life of AVGs and AVFs with effective monitoring, surveillance, and intervention; and using newer technologies to achieve earlier and more consistent AVF and AVG patency to minimize tunneled CVC use. An effective national vascular access quality improvement enterprise will save CMS more money than QIP penalties to providers by decreasing vascular access procedures and hospitalizations, will improve patient QoL, and will avoid placement of the wrong vascular access in the wrong patient at the wrong time.

**Table 1:** Trends in Vascular Access Use in the United States

Year	TCVC >90 d (%)	Total TCVC (%)	AVF (%)	AVG (%)	Data Source
1999	14		26		ESRD CPM
2000	14		27		ESRD CPM
2001	17	21	30	46	ESRD CPM
2002	19	26	31	43	ESRD CPM
2023	21	27	33	41	ESRD CPM
2004	20	27	35	38	ESRD CPM
2005	21	27	39	34	ESRD CPM
2006		29	42	29	FFBI
2007		28	46	26	FFBI
2008		27	49	24	FFBI
2009		26	52	22	FFBI
2010		24	55	21	FFBI
2011		22	58	20	FFBI
2012		20.1	65.8	14.1	DOPPS
2013		16.2	64.2	19.6	DOPPS
2014		16.3	65.2	18.4	DOPPS
2015		16.6	65.5	17.9	DOPPS
2016		15.3	66.6	18.0	DOPPS
2017		13.6	68.4	17.9	DOPPS
2018		12.9	71.6	15.4	DOPPS
2019		15.8	66.5	17.7	DOPPS
2020		17.3	65.0	17.6	DOPPS
2021		19.8	62.5	17.7	DOPPS

Abbreviations: AVF, arteriovenous fistula; AVG, arteriovenous graft; DOPPS, Dialysis Outcomes and Practice Patterns Study (a stratified random sample of US patients receiving kidney replacement therapy, data from https://www.dopps.org/DPM-HD/Files/vType_c_overallTAB.htm using January of each year); ESRD CPM, End Stage Renal Disease Clinical Performance Measures Project (a nationally representative random sample of patients with data gathered during Oct-Dec of the calendar year before the date of the report, individual annual reports available at www.cms.hhs.gov/CPMProject); FFBI, Fistula First Breakthrough Initiative (see text, data from https://www.ncbi.nlm.nih.gov/pmc/articles/PMC5693683/); TCVC, tunneled central venous catheter.
